# Total versus subtotal gastrectomy following neoadjuvant flot chemotherapy for distal diffuse gastric adenocarcinoma: an international cohort study

**DOI:** 10.1007/s10120-026-01746-7

**Published:** 2026-04-27

**Authors:** Jonathan Sivakumar, Darren J. Wong, Katheryn Hall, Margaret M. Lee, Cuong P. Duong, David I. Watson, Claire L. Donohoe, Tim Bright, Ahmad Aly, Kevin Chan, David L. Chan, Neil Merrett, Sivakumar Gananadha, Yick Ho Lam, Harsh Kanhere, BMark Smithers, Michael Bozin, Matthew Read, Krinal Mori, Mary-Ann Johnson, Enoch Wong, Sarah A. Martin, Geraldine Ooi, Yahya Al-Habbal, Chon Hann Liew, Robert Bohmer, Jurstine Daruwalla, Mo Ballal, Rukshan Ranjan, Andrew D. MacCormick, Sharon Pattison, Nicholas Evennett, Jason Robertson, James Tan, Alexandra Gordon, Simon Bann, Inian Samarasam, Ramesh Gurunathan, Jimmy So, Jonathan Yeung, Lorenzo Ferri, Ewen A. Griffiths, Alexander W. Phillips, Sheraz R. Markar, David Chan, Thomas Murphy, John Reynolds, Magnus Nilsson, Guillaume Piessen, Bas Wijnhoven, Richard van Hillegersberg, Mark I. van Berge Henegouwen, Pietro M. Lombardi, SPACE-FLOT Investigators, David S. Liu

**Affiliations:** 1 Division of Cancer Surgery, Peter MacCallum Cancer Centre, Melbourne, VIC 3052 Australia; 2 Department of Surgery, The University of Melbourne, Parkville, Melbourne, VIC 3000 Australia; 3 Department of Gastroenterology, Austin Health, Heidelberg, VIC Australia; 4 Division of Surgery, Anaesthesia and Procedural Medicine, Upper Gastrointestinal Surgery Unit Austin Hospital, Heidelberg, VIC Australia; 5 Department of Surgery, Victorian Interventional Research and Trials Unit, University of Melbourne, Austin Precinct, Heidelberg, VIC Australia; 6 Department of Medical Oncology, Box Hill Hospital, Box Hill, VIC Australia; 7 College of Medicine and Public Health, Flinders University, Flinders, South Australia Australia; 8 Department of Surgery, Flinders Medical Centre, Flinders, South Australia Australia; 9 Upper Gastrointestinal Surgery Unit, St James Hospital, Dublin, Ireland; 10 Upper Gastrointestinal Surgery Unit, Royal Brisbane and Women’s Hospital, Herston, QLD Australia; 11 Upper Gastrointestinal Surgery Unit, Royal Northshore Hospital, St Leonards, New South Wales Australia; 12 Northern Clinical School, Faculty of Medicine and Health, University of Sydney, Sydney, NSW Australia; 13 Upper Gastrointestinal Surgical Unit, Bankstown-Lidcombe Hospital, Bankstown, NSW Australia; 14 School of Medicine, Western Sydney University, Campbelltown, NSW Australia; 15 Upper Gastrointestinal Surgery Unit, Canberra Hospital, Canberra, Australia; 16 Upper Gastrointestinal Surgery Unit, Lyell McEwin Hospital, Adelaide, South Australia Australia; 17 Upper Gastrointestinal Surgery Unit, Royal Adelaide Hospital, Adelaide, South Australia Australia; 18 Upper Gastrointestinal Surgery Unit, Princess Alexandra Hospital, University of Queensland, Brisbane, Australia; 19 Upper Gastrointestinal Surgery Unit, St Vincent’s Hospital, Fitzroy, VIC Australia; 20 Upper Gastrointestinal Surgery Unit, Northern Hospital, Epping, VIC Australia; 21 Upper Gastrointestinal Surgery Unit, Monash Medical Centre, Epping, VIC Australia; 22 Upper Gastrointestinal Surgery Unit, Western Health, Footscray, VIC Australia; 23 Upper Gastrointestinal Surgery Unit, Bendigo Health, Bendigo, VIC Australia; 24 Upper Gastrointestinal Surgery Unit, Royal Hobart Hospital, Hobart, TAS Australia; 25 Upper Gastrointestinal Surgery Unit, Launceston General Hospital, Launceston, TAS Australia; 26 Upper Gastrointestinal Surgery Unit, Fiona Standley Hospital, Perth, WA Australia; 27 Upper Gastrointestinal Surgery Unit, Christchurch Hospital, Christchurch, New Zealand; 28 Upper Gastrointestinal Surgery Unit, Middlemore Hospital, Auckland, New Zealand; 29 Southern Blood and Cancer Service, Health New Zealand Te Whatu Ora—Southern, Auckland, New Zealand; 30 Upper Gastrointestinal Surgery Unit, Auckland City Hospital, Auckland, New Zealand; 31 Upper Gastrointestinal Surgery Unit, North Shore Hospital, Auckland, New Zealand; 32 Upper Gastrointestinal Surgery Unit, Palmerston North Hospital, Palmerston, New Zealand; 33 Upper Gastrointestinal Surgery Unit, Wellington Regional Hospital, Wellington, New Zealand; 34 Department of Surgery, Monash University, Melbourne, VIC Australia; 35 Upper Gastrointestinal Surgery Unit, Box Hill Hospital, Box Hill, VIC Australia; 36 Department of Pathology, Otago Medical School—Dunedin Campus, University of Otago Ōtākou Whakaihu Waka, Dunedin, New Zealand; 37 Department of Surgery, University of Auckland, Auckland, New Zealand; 38 Upper Gastrointestinal Surgery Unit, Christian Medical College Hospital, Vellore, India; 39 Cengild G.I. Medical Center, Kuala Lumpur, Malaysia; 40 Division of General Surgery, Department of Surgery, National University Hospital, Singapore, Singapore; 41 Division of Surgical Oncology, National University Cancer Institute, Singapore, Singapore; 42 Yong Loo Lin School of Medicine, National University of Singapore, Singapore, Singapore; 43 Department of Surgery, Toronto General Hospital, Toronto, Canada; 44 Division of Thoracic and Upper Gastrointestinal Surgery, McGill University Health Centre, Montreal, Canada; 45 Department of Upper GI Surgery, Queen Elizabeth Hospital, Birmingham, England; 46 Northern Oesophagogastric Unit, Royal Victoria Infirmary, Newcastle Upon Tyne, England; 47 Surgical Interventional Trials Unit, Nuffield Department of Surgical Sciences, Oxford University Hospital, London, England; 48 Department of Surgery, University Hospitals Plymouth, Plymouth, England; 49 Department of Surgery, Mercy University Hospital, Cork, Ireland; 50 Karolinska University Hospital and CLINTEC Karolinska Institutet, Stockholm, Sweden; 51 Department of Digestive and Oncological Surgery, Lille University Hospital, Lille, France; 52 Department of Surgery, Erasmus University Medical Center, Rotterdam, Netherlands; 53 Department of Surgery, University Medical Centre Utrecht, Utrecht, Netherlands; 54 Department of Surgery, Amsterdam UMC, Amsterdam, Netherlands; 55 Surgical Oncology, Niguarda Cancer Center, Milan, Italy; 56 Institute of Immunology and Immunotherapy, University of Birmingham, Birmingham, England

**Keywords:** Diffuse gastric cancer, Gastrectomy

## Abstract

**Objective:**

To compare perioperative, oncological, and survival outcomes of total gastrectomy (TG) versus subtotal gastrectomy (SG) in patients with locally advanced distal diffuse gastric adenocarcinoma treated with perioperative 5-fluorouracil, leucovorin, oxaliplatin and docetaxel (FLOT) chemotherapy.

**Background:**

Diffuse distal gastric cancer is characterized by infiltrative growth patterns and early nodal metastasis. Whilst radical resection remains the cornerstone of curative treatment, the optimal extent of surgery with TG or SG, remains debated.

**Methods:**

This international multicenter cohort study analyzed data from patients with histologically confirmed diffuse gastric adenocarcinoma, located > 5 cm from the gastroesophageal junction. Endpoints included surgical margin status, nodal yield, perioperative morbidity, recurrence patterns, time-to-recurrence (TTR), and overall survival (OS). Outcomes were compared using multivariate analyses.

**Results:**

In total, 188 (39.0%) patients underwent TG and 294 (61.0%) underwent SG. After multivariable adjustment, surgical margin positivity was comparable between groups (OR 1.28, 95%CI 0.70–2.34). TG was associated with higher total nodal yield [Median(IQR) 31 (23–41) vs 28 (18–36), p < 0.001] but not metastatic nodal yield [Median(IQR) 1 (0–8) vs 1 (0–6), p = 0.065]. TG had longer operative time [Mean(SD) 318.5 (93.6) vs 301.0 (105.2) minutes, p = 0.040], extended hospital stay [Median(IQR) 8.5 (7.0–11.0) vs 7.0 (6.0–9.0), p < 0.001], and more complications (OR 1.55, 95%CI 1.05–2.30). Recurrence patterns and adjusted TTR (HR 1.29, 95%CI 0.95–1.75) were similar between groups. Adjusted OS was superior in the SG group (HR 1.69, 95%CI 1.20–2.38).

**Conclusions:**

In appropriately selected patients, SG has comparable oncological efficacy to TG with lower surgical morbidity for distal diffuse gastric adenocarcinoma post FLOT chemotherapy.

**Supplementary Information:**

The online version contains supplementary material available at 10.1007/s10120-026-01746-7.

## Introduction

Locally advanced gastric adenocarcinoma is common, with surgical resection and perioperative chemotherapy being the cornerstone of curative-intent treatment in Western countries [1–3]. The Lauren classification distinguishes gastric adenocarcinoma into intestinal and diffuse subtypes [4–6]. Diffuse gastric cancer, which comprises one third of all cases, carries a unique set of therapeutic challenges [7, 8]. This disease is characterized by poorly cohesive cellular growth patterns with a predilection for extensive submucosal infiltration and early nodal metastasis [4, 9]. These features form the premise for a more extensive resection to ensure clear surgical margins and adequate lymphadenectomy, influencing the tendency to favor total gastrectomy for these aggressive tumors.

For distal gastric cancers located more than 5 cm below the gastroesophageal junction, the ESMO 2022 Clinical Practice Guidelines suggest that a subtotal rather than total gastrectomy will suffice [1]. However, for diffuse gastric cancers, substantial practice variation persists due to the fear of positive resection margins and inadequate lymphadenectomy. A survey of 62 European centers reported that 44% of surgeons would routinely perform a total gastrectomy for a diffuse gastric cancer, irrespective of anatomical location or their ability to obtain adequate surgical margins [10]. While this debate has persisted for many years [11–13], the therapeutic landscape for gastric cancers has undergone a transformation with the introduction of modern chemotherapy. The landmark FLOT4-AIO trial demonstrated that perioperative FLOT (5- fluorouracil, leucovorin, oxaliplatin, docetaxel) chemotherapy significantly improved overall survival for locally advanced gastric cancer compared to older regimens, achieving higher rates of disease regression in the primary tumor and nodal stations [14, 15].

The adoption of FLOT as the standard of care has raised questions about the optimal extent of resection for distal diffuse gastric cancer. Moreover, contemporary surgical philosophy does increasingly favor organ-conserving approaches when oncologically appropriate. This is supported by a growing body of evidence suggesting that subtotal gastrectomy offers superior quality-of-life outcomes when compared with total gastrectomy [16–18]. However, there is currently no evidence comparing the extent of resections for distal diffuse gastric cancer in patients treated with perioperative FLOT chemotherapy.

The SPACE-FLOT (Survival and Patterns of Care in the Era of FLOT-based chemotherapy for gastro-oesophageal cancers) international multicenter cohort study offers a unique opportunity to address this important knowledge gap [19]. Accordingly, the aim of this study is to compare the perioperative, oncological and survival outcomes of patients who underwent total (TG) versus subtotal (SG) gastrectomy for distal diffuse gastric adenocarcinoma in the setting of perioperative FLOT chemotherapy.

## Methods

### Study design

This is an international, multicenter cohort study conducted within the SPACE-FLOT collaborative network [19]. Data was retrospectively collected from consecutive patients recorded in prospectively maintained databases from 43 participating centers in 12 countries (Table S1). The study period spanned from 2017 to 2022, corresponding to the widespread adoption of perioperative FLOT chemotherapy. Ethical approval was obtained from the Peter MacCallum Cancer Centre Human Research Ethics Committee (HREC/76492/PMCC) and from local ethics committees at all participating sites, in accordance with the Declaration of Helsinki [20]. This study was registered with the Australian New Zealand Clinical Trials Registry (ACTRN12622000180718).

### Patient population

Eligible patients had histologically confirmed diffuse gastric adenocarcinoma as per WHO classification [21], received neoadjuvant FLOT chemotherapy, and underwent radical gastric resection. Only tumors located in the gastric body, antrum, or pre-pyloric region, with a minimum of 5 cm clearance from the gastroesophageal junction (top of the gastric folds) to the macroscopically visible proximal tumor edge, were included to ensure comparability of resection strategies. Patients were excluded if they had macroscopically visible cancer within 5 cm of the gastroesophageal junction, intestinal subtype, non-adenocarcinoma histology, non-regional nodal metastases, or distant metastases, including peritoneal carcinomatosis or positive peritoneal cytology. Patients below the age of 18 years were also excluded. Standardized FLOT chemotherapy consisted of 5-fluorouracil, leucovorin, oxaliplatin, and docetaxel, was administered every two weeks for a maximum of eight perioperative cycles as per patient tolerance and clinician discretion [14, 19].

### Surgery

The surgical approach, extent of gastric and nodal resection, as well as reconstruction technique was determined by the treating surgical team. TG involved complete removal of the stomach with transection points across the duodenum and distal esophagus. SG involved distal transection across the duodenum and proximal gastric transection within 5 cm below the gastroesophageal junction. All surgeries were performed with the aim of achieving clear resection margins according to international guidelines [1]. A concurrent radical lymphadenectomy removing at least gastric nodal stations 1, 3 to 7 for SG and stations 1 to 7 for TG was also performed (Table 1) [22].

**Table 1:** Baseline characteristics

Characteristics	Total gastrectomy (n = 188)	Subtotal gastrectomy (n = 294)	p-value
Demographics			
Age (years), mean (SD)	58.7 (11.8)	60.0 (11.9)	0.239
Sex (male), n (%)	99 (52.7)	178 (60.5)	0.090
BMI (kg/m^2^), mean (SD)	25.9 (5.4)	25.7 (4.5)	0.723
Charlson Co-morbidity Index			0.064
Index score, median (IQR)	2 (1–3)	2 (1–3)	
Predicted 10-year survival (%), mean (SD)	82.5 (22.3)	78.2 (25.7)	
Smoking status at time of surgery, n (%)			0.506
Active	28 (14.9)	45 (15.3)	
Former	58 (30.9)	82 (27.9)	
Never	73 (38.8)	106 (36.1)	
Unknown	29 (15.4)	61 (20.7)	
ASA grade at time of surgery, n (%)			0.243
1	20 (10.6)	41 (13.9)	
2	108 (57.4)	134 (45.6)	
3	59 (31.4)	114 (38.8)	
4	1 (0.5)	5 (1.7)	
ECOG grade at time of surgery, n (%)			0.219
0	120 (63.8)	172 (58.5)	
1	64 (34.0)	112 (38.1)	
2	3 (1.6)	9 (3.1)	
3	1 (0.5)	1 (0.3)	
Neoadjuvant treatment			
Completed 4 cycles of neoadjuvant FLOT, n (%)	149 (79.3)	251 (85.4)	0.100
FLOT cycles completed, median (IQR)	4 (4–4)	4 (4–4)	0.112
Clinical tumor features			
Anatomical location of tumor, n (%)			0.001
Gastric body-antrum region	181 (96.3)	254 (86.4)	
Gastric pre-pyloric region	7 (3.7)	40 (13.6)	
Clinical stage, n (%)			0.118
cT1 N0	11 (5.9)	17 (5.8)	
cT2-3 N0	77 (41.0)	141 (48.0)	
cT4a N0	20 (10.6)	19 (6.5)	
cT4b N0	0 (0.0)	2 (0.7)	
cT1 N1	5 (2.7)	2 (0.7)	
cT2-3 N1	55 (29.3)	91 (31.0)	
cT4a N1	20 (10.6)	20 (6.8)	
cT4b N1	0 (0.0)	2 (0.7)	
Surgery details			
Operative approach, n (%)			0.082
Minimally invasive	128 (68.1)	176 (59.9)	
Open	60 (31.9)	118 (40.1)	
Type of lymphadenectomy, n (%)			0.376
D1	10 (5.3)	19 (6.5)	
D1 +	68 (36.2)	122 (41.5)	
D2	110 (58.5)	153 (52.0)	
Tumor histology			
Lymphovascular invasion, n (%)	89 (47.3)	115 (39.1)	0.089
Perineural invasion, n (%)	86 (45.7)	106 (36.1)	0.036
Tumor Regression Grading, n (%)			0.258
Complete responders	12 (6.4)	26 (8.8)	
Partial responders	116 (61.7)	160 (54.4)	
Minimal responders	60 (31.9)	108 (36.7)	
Pathological stage, n (%)			0.219
1	39 (20.7)	87 (29.6)	
2	26 (13.8)	36 (12.2)	
3a	7 (3.7)	15 (5.1)	
3b	46 (24.5)	59 (20.1)	
4a	70 (37.2)	97 (33.0)	

ASA, American society of anesthesiologists; BMI, body mass index; ECOG, eastern cooperative oncology group performance status grading; IQR, interquartile range; SD, standard deviation

### Data collection

Data was collected through an online REDCap database that was designed, tested and validated by the Peter MacCallum Data Systems Research Computing Facility. Quality assurance measures included standardized data dictionary, training sessions for data collectors, in-program data field prompts, real-time data entry support, central data cleaning by two independent investigators, and random auditing of 10% of records to verify accuracy against source documentation, with overall data concordance exceeding 95% [mean (SD), 97.8 (2.3)%].

### Study outcomes and definitions

The endpoints of this study included surgical margin status, histologically proven total and positive/metastatic nodal yield, operative duration, incidence of intraoperative complications, length of hospital stay, 30-day perioperative outcomes, rates of starting and completing adjuvant treatment, postoperative complication profile, pattern of cancer recurrence, time to recurrence (TTR), and overall survival (OS). R0 resection margin was defined as no microscopic (R1) or macroscopic (R2) tumor involvement at the proximal, distal and circumferential transection edges. TTR was calculated from the date of surgery to the date of disease recurrence as determined by clinical, endoscopic, and/or radiological examinations. OS was determined as the interval from surgery to death from any cause. Those alive at study termination were censored at the time of last contact.

Baseline comorbidities were quantified using the Charlson Comorbidity Index, which also estimates the probability of survival at 10 years.[23] Clinicians’ assessment of performance status was classified using the Eastern Cooperative Oncology Group (ECOG) scale.[24] Clinicians’ assessment of overall surgical fitness were classified using the American Society of Anesthesiologist (ASA) score.[25] Postoperative medical complications were defined according to the European Perioperative Clinical Outcome definitions.[26] Postoperative surgical complications were defined by the Esophagectomy Complication Consensus Guidelines.[27] The severity of each adverse event was classified using the Clavien-Dindo system.[28] All tumors were staged using the 8th Edition AJCC Cancer Staging Manual [29].

### Statistical analysis

Univariate comparisons between study groups were performed using Fisher’s exact test and Student’s t test for categorical and continuous variables respectively. For non-parametric continuous data, the Mann–Whitney U test was used. Where comparisons involved more than two categorical variables, the chi-square test was applied. Multivariate logistic regression (for categorical variables) or Poisson regression (for continuous variables) analyses were performed to adjust for confounding effects of baseline characteristics (Table 1) on surgical margins, nodal yield, and perioperative outcomes (Table 3). Baseline variables with a *p* < 0.2 on univariate comparison were input into multivariate modelling. Unadjusted TTR and OS were analyzed using log rank test. Multivariate Cox proportional hazards models were fitted adjusting for age, gender, body mass index, Charlson Comorbidity Index, smoking status, ASA score, ECOG grade, tumor location (body, antrum, pre-pylorus), completion of neoadjuvant FLOT chemotherapy, extent of lymphadenectomy, presence of lymphovascular infiltration and perineural invasion, pathological tumor stage, tumor regression grading, and receipt of adjuvant chemotherapy. The proportional hazards assumption was tested for all models. Two-tail *P* values < 0.050 and odds (OR) or hazards (HR) ratio with 95% confidence intervals that did not cross one were considered statistically significant. Statistical analyses were undertaken using Prism version 10.2.2 (GraphPad Software, San Diego, CA, USA) and R version 4.1.0 (R Foundation for Statistical Computing, Vienna, Austria).

## Results

### Baseline characteristics

In total, 482 patients were eligible, of whom 188 (39.0%) underwent TG and 294 (61.0%) underwent SG. This represents 21.4% of the SPACE-FLOT dataset. Detailed study population characteristics are shown in Table 1. The mean age of patients was 59.5 (SD 11.9) years and 277 (57.5%) were male. A total of 454 (94.2%) had locally advanced disease (cT ≥ 2 and/or cN1 +) located in the distal stomach. Overall, 400 (83.0%) patients completed all 4 cycles of neoadjuvant FLOT and 371 (77.0%) received adjuvant chemotherapy. The median follow-up duration was 26.4 (IQR 14.7–39.9) months post-surgery. Besides a higher rate of pre-pyloric tumors in SG group and a higher rate of perineural invasion in the TG group, baseline demographics, perioperative treatment, surgery, and clinicopathological tumor characteristics were similar between study groups.

### Surgical margins

Following multivariate analysis, TG and SG groups were comparable with respect to their rate of positive (R1 or R2) proximal (OR 2.16, 95% CI 0.96–5.08, p = 0.068), distal (OR 1.20, 95% CI 0.50–2.86, p = 0.681), and circumferential (OR 0.99 95% CI 0.45–2.16, p = 0.979) resection margins (Table 2). Further analysis of proximal margin status comparing TG and SG groups revealed 1) no cases of macroscopically (R2) involved margins, 2) higher rate of microscopically (R1) involved margins for tumors located in the gastric body than in the antrum or pre-pyloric regions regardless of the extent of surgical resection, and 3) similar risk of proximal margin-positivity segregated by tumor location within the stomach (Table S2).

**Table 2:** Surgical margins, nodal and perioperative outcomes

Outcomes	Total gastrectomy (n = 188)	Subtotal gastrectomy (n = 294)	Unadjusted cohort		Adjusted*	
			OR (95% CI)	p-value	OR (95% CI)	p-value
Positive surgical margin (R1 or R2), n (%)						
Any margin	28 (14.9)	31 (10.5)	1.49 (0.84–2.57)	0.158	1.28 (0.70–2.34)	0.413
Proximal margin	17 (9.0)	11 (3.7)	2.56 (1.18–5.82)	0.026	2.16 (0.96–5.08)	0.068
Distal margin	11 (5.9)	14 (4.8)	1.24 (0.56–2.70)	0.675	1.20 (0.50–2.86)	0.681
Circumferential margin	14 (7.4)	19 (6.5)	1.17 (0.56–2.41)	0.713	0.99 (0.45–2.16)	0.979
Nodal yield, median (IQR)						
All harvested nodes	31 (23–41)	28 (18–36)	-	0.002	-	< 0.001
Positive/metastatic nodes	1 (0–8)	1 (0–6)	-	0.226	-	0.065
Perioperative outcomes						
Duration of surgery, mean (SD)	318.5 (93.6)	301.0 (105.2)	-	0.035	-	0.040
Intraoperative complications, n (%)	9 (4.8)	14 (4.8)	1.01 (0.41–2.32)	1.000	1.07 (0.42–2.64)	0.880
Length of hospital stay, median (IQR)	8.5 (7.0–11.0)	7.0 (6.0–9.0)	-	< 0.001	-	< 0.001
30-day return to theatre, n (%)	17 (9.0)	25 (8.5)	1.07 (0.56–2.02)	0.869	1.16 (0.58–2.27)	0.675
30-day hospital re-admission, n (%)	16 (8.5)	25 (8.5)	1.00 (0.51–1.92)	1.000	0.93 (0.47–1.82)	0.844
30-day ICU readmission, n (%)	11 (5.9)	7 (2.4)	2.55 (0.99–6.58)	0.082	2.88 (1.07–8.40)	0.041
30-day mortality, n (%)	0 (0.0)	0 (0.0)	-	1.000	-	1.000
Starting adjuvant treatment, n (%)	142 (75.5)	229 (77.9)	0.88 (0.57–1.34)	0.580	0.77 (0.49–1.23)	0.271
Completing adjuvant treatment, n (%)	98 (52.1)	170 (57.8)	0.79 (0.54–1.12)	0.224	0.86 (0.48–1.53)	0.594

CI, confidence interval; IQR, interquartile range; OR, odds ratio, R1: microscopic involved margins; R2: macroscopically involved margins; SD, Standard deviation.

* Adjusted using multivariate logistic regression (for categorical variables) or Poisson regression (for continuous variables) based on variables from Table 1 with a univariate p < 0.2. See Table S2 for additional information

### Nodal yield

Despite a significantly higher number of lymph nodes harvested in the TG group [median (IQR) 31 [23–41] vs 28 [18–36], adjusted p < 0.001], the burden of positive nodal metastasis was comparable between groups [median (IQR) 1 (0–8) vs 1 (0–6), adjusted p = 0.065] (Table 2).

### Perioperative outcomes

Key perioperative outcomes are summarized in Table 2. Following multivariate analysis, compared with the SG group, TG was associated with longer operative duration [mean (SD) 318.5 (93.6) vs 301.0 (105.2) minutes, *p* = 0.040], longer length of hospital stay [median (IQR) 8.5 (7.0–11.0) vs 7.0 (6.0–9.0) days, *p* < 0.001], and a higher rate of unplanned 30-day ICU readmission (OR 2.88, 95% CI 1.07–8.40, *p* = 0.041).

### Surgical complication profile

As detailed in Table 3, based on ECCG and Clavien-Dindo grading, patients who underwent TG had a higher rate of major complications (OR 1.55, 95% CI 1.05–2.30, *p* = 0.028) compared with those who underwent SG. Specifically, there was a significantly higher rate of pneumonia (12.2% vs 7.1%, *p* = 0.043), cardiac arrhythmias (7.4% vs 2.0%, *p* = 0.005), wound infection (5.9% vs 2.0%, *p* = 0.040) and *Clostridium difficile* infection (2.7% vs 0.3%, *p* = 0.036) within the TG group.

**Table 3:** Surgical complication profile

Complications	Total gastrectomy (n = 188)	Subtotal gastrectomy (n = 294)	OR (95% CI)	p-value
Clavien-Dindo complication grade, n (%)			-	0.068
No complications	106 (56.4)	188 (63.9)		
Grade 1	10 (5.3)	22 (7.5)		
Grade 2	47 (25.0)	51 (17.3)		
Grade 3 a/b	18 (9.6)	25 (8.5)		
Grade 4 a/b	7 (3.7)	8 (2.7)		
Grade 5	0 (0.0)	0 (0.0)		
Clavien-Dindo Grade ≥ 2	72 (38.3)	84 (28.6)	1.55 (1.05–2.30)	0.028
Pulmonary, n (%)				
Pneumonia	23 (12.2)	21 (7.1)	1.81 (1.00–3.40)	0.043
Pleural effusion requiring drainage	5 (2.7)	2 (0.7)	3.99 (0.85–20.18)	0.116
Respiratory failure requiring intubation	2 (1.1)	2 (0.7)	1.57 (0.24–10.08)	0.645
Pneumothorax requiring intervention	0 (0.0)	2 (0.7)	0.00 (0.00–3.38)	0.523
Acute respiratory distress syndrome	1 (0.5)	2 (0.7)	0.78 (0.05–6.76)	1.000
Acute aspiration	2 (1.1)	0 (0.0)	-	0.152
Atelectasis requiring bronchoscopy	0 (0.0)	2 (0.7)	-	0.523
Cardiac, n (%)				
Arrhythmias requiring intervention	14 (7.4)	6 (2.0)	3.86 (1.43–9.89)	0.005
Congestive heart failure requiring intervention	2 (1.1)	1 (0.3)	3.15 (0.36–45.79)	0.563
Myocardial infarction	2 (1.1)	1 (0.3)	3.15 (0.36–45.79)	0.563
Cardiac arrest requiring intervention	0 (0.0)	0 (0.0)	-	1.000
Pericarditis requiring treatment	0 (0.0)	0 (0.0)	-	1.000
Gastrointestinal, n (%)				
Anastomotic leak	14 (7.4)	17 (5.8)	1.31 (0.61–2.64)	0.569
Ileus delaying enteral feeding	7 (3.7)	15 (5.1)	0.72 (0.27–1.76)	0.655
Small bowel obstruction	7 (3.7)	4 (1.4)	2.80 (0.88–8.65)	0.119
Chyle leak	3 (1.6)	4 (1.4)	1.18 (0.29–4.42)	1.000
Pancreatitis	0 (0.0)	0 (0.0)	-	1.000
Liver dysfunction	2 (1.1)	3 (1.0)	1.04 (0.18–5.14)	1.000
Infection, n (%)				
General sepsis	7 (3.7)	8 (2.7)	1.38 (0.52–3.87)	0.595
Surgical site infection requiring intervention	11 (5.9)	6 (2.0)	2.98 (1.09–8.03)	0.040
Clostridium difficile infection	5 (2.7)	1 (0.3)	8.01 (1.09–94.63)	0.036
Line infection requiring intervention	1 (0.5)	4 (1.4)	0.39 (0.03–2.36)	0.653
Intrathoracic abscess	1 (0.5)	2 (0.7)	0.78 (0.05–6.76)	1.000
Intraabdominal abscess	9 (4.8)	12 (4.1)	1.18 (0.49–2.77)	0.820
Neurological, n (%)				
Delirium	2 (1.1)	2 (0.7)	1.57 (0.24–10.08)	0.645
Cerebrovascular accident	0 (0.0)	0 (0.0)	-	1.000
Hematological, n (%)				
Bleeding requiring intervention	6 (3.2)	10 (3.4)	0.94 (0.34–2.58)	1.000
Venous thromboembolism	1 (0.5)	4 (1.4)	0.39 (0.03–2.36)	0.653
Urological, n (%)				
Acute kidney injury	0 (0.0)	3 (1.0)	0.00 (0.00–1.80)	0.285
Urinary tract infection	7 (3.7)	7 (2.4)	1.59 (0.59–4.23)	0.415
Skin, n (%)				
Wound dehiscence	1 (0.5)	4 (1.4)	0.39 (0.03–2.36)	0.653
Acute abdominal wall hernia	0 (0.0)	2 (1.1)	0.00 (0.00–3.38)	0.523

CI: confidence interval, IQR: interquartile range, OR: odds ratio, SD: standard deviation

### Oncological outcomes

**Fig. 1:**
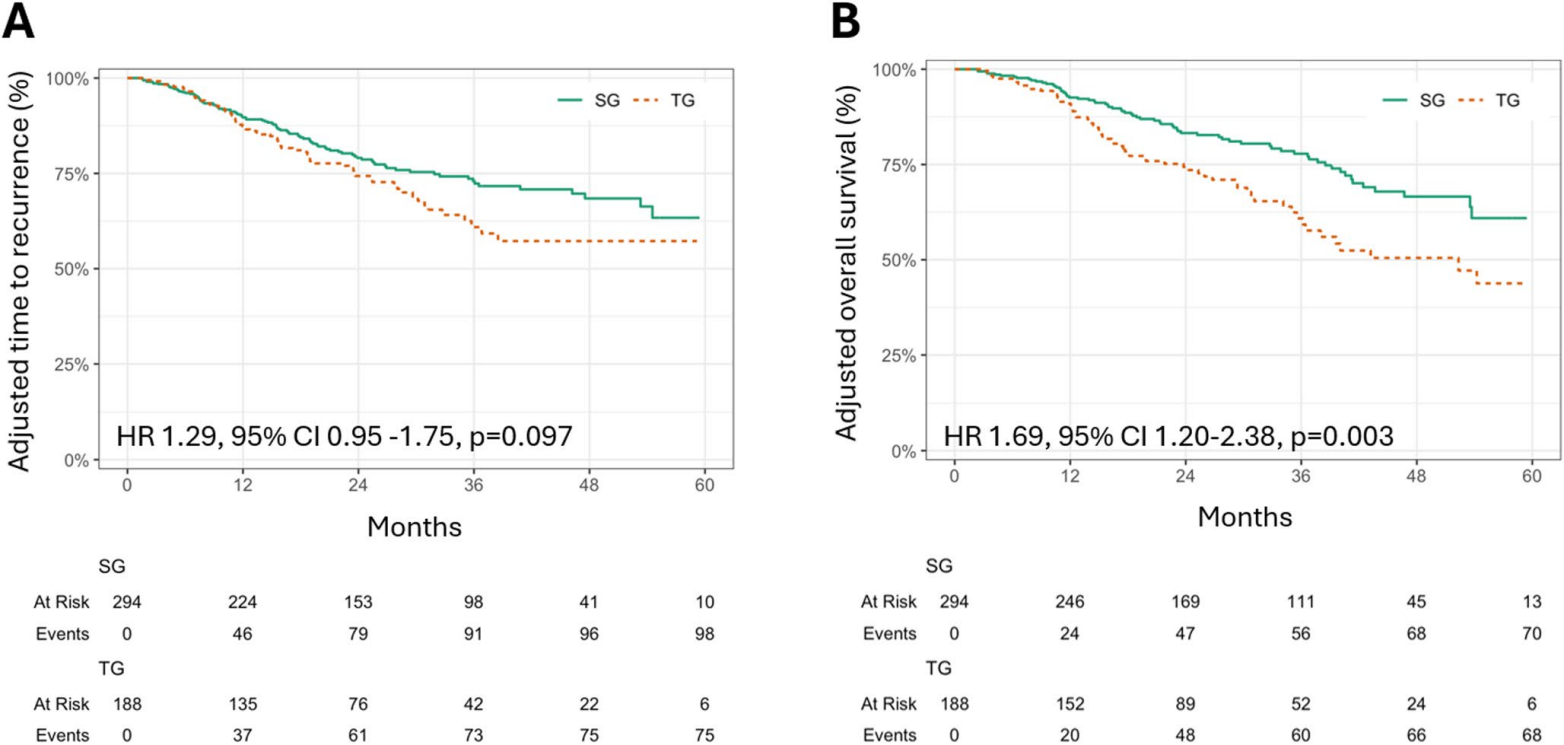
Comparison of patients with diffuse distal gastric adenocarcinoma undergoing TG versus STG with respect to **A** adjusted time to recurrence and **B** overall survival. TG, Total gastrectomy; SG, Subtotal gastrectomy; HR, Hazard ratio; CI, Confidence interval

Oncologic follow-up demonstrated broadly similar patterns of cancer recurrence between TG and SG groups (Figure S1). Importantly, there was no difference between the two groups with respect to locoregional recurrence. Among patients who developed disease relapse, the peritoneal cavity was the most common site of first recurrence. This was observed in 20.7% of cases. Extent of gastric resection (TG vs SG) did not affect likelihood of peritoneal disease. Following Cox regression analysis (Fig. 1), TTR was comparable between TG and SG groups (Adjusted HR 1.29, 95% CI 0.95–1.75, p = 0.097). Interestingly, patients who underwent TG had poorer OS (Adjusted HR 1.69, 95% CI 1.20–2.38, *p* = 0.003) compared to the SG group. Detailed univariate and multivariate Cox regression analyses for TTR and OS (Tables 4 and 5) and unadjusted survival curves (Figure S2) are provided in supplementary materials.

**Table 4:** Univariate and multivariate cox regression analysis for time to recurrence

Characteristics	Univariate			Multivariate		
	HR	95% CI	p-value	HR	95% CI	p-value
Demographics						
Age (years)	0.99	0.97–1.00	0.022	0.99	0.98–1.01	0.539
Sex (male)	1.02	0.75–1.38	0.906	–	–	–
BMI (kg/m^2^)	0.94	0.91–0.97	< 0.001	0.95	0.92–0.98	< 0.001
Charlson Co-morbidity Index (score)	0.92	0.84–1.01	0.084	1.00	0.88–1.13	0.995
Smoking status at time of surgery						
Never	Ref	Ref				
Former	1.28	0.88–1.86	0.190	1.38	0.93–2.06	0.112
Active	1.46	0.96–2.24	0.078	1.22	0.79–1.90	0.371
Unknown	0.88	0.56–1.39	0.593	1.36	0.83–2.22	0.219
ASA grade at time of surgery (grade)	0.97	0.78–1.19	0.756	–	–	–
ECOG grade at time of surgery (grade)	0.77	0.58–1.02	0.069	0.82	0.62 – 1.09	0.178
Perioperative treatment						
Neoadjuvant FLOT treatment completed (yes)	1.21	0.80–1.81	0.363	–	–	–
Adjuvant FLOT treatment (yes)	0.95	0.66–1.35	0.766	–	–	–
Clinical tumor features						
Anatomical location of tumor						
Gastric pre-pyloric region	Ref	–	–	–		
Gastric body-antrum region	0.83	0.52 – 1.32	0.420	–	–	–
Surgery details						
Gastrectomy approach						
Subtotal	Ref	Ref				
Total	1.35	1.00–1.82	0.051	1.29	0.95–1.75	0.097
Operative approach						
Open	Ref	Ref				
Minimally invasive	0.76	0.55–1.04	0.085	0.97	0.69–1.36	0.850
Type of lymphadenectomy						
D1	Ref	Ref				
D1 +	1.45	0.58–3.63	0.423	1.25	0.48–3.25	0.647
D2	2.29	0.93–5.61	0.070	1.65	0.64–4.20	0.298
Tumor histology						
Lymphovascular invasion (yes)	2.91	2.05–4.13	< 0.001	1.02	0.68–1.51	0.850
Perineural invasion (yes)	2.72	1.92–3.86	< 0.001	1.16	0.78–1.72	0.458
Tumor Regression Grading						
Complete responders	Ref	Ref				
Partial responders	13.7	1.91–98.20	0.009	0.62	0.06 – 7.08	0.704
Minimal responders	23.4	3.26–168.0	0.002	0.78	0.07–9.00	0.844
Surgical margin status						
Negative (R0)	Ref	Ref				
Positive (R1 or R2)	2.55	1.79–3.63	< 0.001	1.23	0.83–1.82	0.298
Pathological stage						
1	Ref	Ref				
2	8.82	2.51–30.90	< 0.001	7.79	1.74–35.00	0.007
3	19.8	6.18–63.70	< 0.001	17.9	4.27–75.30	< 0.001
4	46.4	14.7–146.00	< 0.001	39.1	9.32–164.00	< 0.001

ASA, American society of anesthesiologists; BMI, body mass index; CI, confidence interval; ECOG, eastern cooperative oncology group performance status grading; HR, hazard ratio

**Table 5:** Univariate and multivariate cox regression analysis for overall survival

Characteristics	Univariate			Multivariate		
	HR	95% CI	p-value	HR	95% CI	p-value
Demographics						
Age (years)	1.00	0.99–1.02	0.876	–	–	–
Sex (male)	1.01	0.72–1.41	0.962	–	–	–
BMI (kg/m^2^)	0.97	0.94–1.01	0.116	0.98	0.95 – 1.02	0.296
Charlson Co-morbidity Index (score)	1.01	0.92–1.11	0.829	–	–	–
Smoking status at time of surgery						
Never	Ref	Ref				
Former	1.77	1.15–2.73	0.010	1.70	1.08 – 2.67	0.022
Active	1.78	1.08–2.93	0.023	1.50	0.89 – 2.43	0.129
Unknown	1.41	0.87–2.29	0.167	1.60	0.96 – 2.69	0.073
ASA grade at time of surgery (grade)	1.13	0.90–1.43	0.300	–	–	–
ECOG grade at time of surgery (grade)	0.89	0.65–1.21	0.445	–	–	–
Perioperative treatment						
Neoadjuvant FLOT treatment completed (yes)	1.06	0.69–1.64	0.798	–	–	–
Adjuvant FLOT treatment (yes)	0.74	0.51–1.08	0.121	0.70	0.47 – 1.04	0.080
Clinical tumor features						
Anatomical location of tumor						
Gastric pre-pyloric region	Ref	–	–	–		
Gastric body-antrum region	0.86	0.51–1.45	0.574	–	–	–
Surgery details						
Gastrectomy approach						
Subtotal	Ref	Ref				
Total	1.72	1.23–2.40	0.001	1.69	1.20, 2.38	0.003
Operative approach						
Open	Ref	Ref				
Minimally invasive	0.79	0.56–1.12	0.186	0.98	0.67 – 1.44	0.933
Type of lymphadenectomy						
D1	Ref	–	–	–		
D1 +	0.88	0.40–1.94	0.748	–	–	–
D2	1.20	0.55–2.59	0.650	–	–	–
Tumor histology						
Lymphovascular invasion (yes)	3.95	2.59–6.02	< 0.001	1.39	0.85 – 2.27	0.189
Perineural invasion (yes)	3.42	2.25–5.19	< 0.001	1.41	0.87–2.28	0.163
Tumor Regression Grading						
Complete responders	Ref	Ref				
Partial responders	10.3	1.44–74.4	0.020	1.85	0.21–16.3	0.682
Minimal responders	16.7	2.32–120	0.005	1.96	0.22–17.6	0.549
Surgical margin status						
Negative (R0)	Ref	Ref				
Positive (R1 or R2)	2.70	1.84–3.96	< 0.001	1.26	0.83–1.91	0.285
Pathological stage						
1	Ref	Ref				
2	3.43	1.25–9.44	0.017	2.61	0.88–7.78	0.085
3	6.89	2.89–16.4	< 0.001	4.96	1.87–13.1	0.001
4	17.2	7.51–39.4	< 0.001	11.2	4.26–29.7	< 0.001

ASA, American society of anesthesiologists; BMI, body mass index; CI, confidence interval; ECOG, eastern cooperative oncology group performance status grading; HR, hazard ratio

## Discussion

This international multicenter cohort study represents the largest analysis comparing TG versus SG for distal diffuse gastric adenocarcinoma in the era of perioperative FLOT chemotherapy. Our key findings demonstrate that in appropriately selected patient cohorts, SG achieves comparable oncological outcomes as TG but with reduced perioperative morbidity.

Our findings largely align with evidence from the pre-FLOT era questioning the need for routine TG for all diffuse gastric cancers. One of the earliest studies to evaluate the impact of resection strategy for distal diffuse gastric cancer was conducted by Roukos et al. [30], who found that amongst 39 patients evaluated, TG conferred no survival advantage over SG, with a 5-year OS of 57.7% in the TG group versus 65.8% in the SG group. Similarly, Arer et al. analyzed 46 patients with signet ring cell distal gastric cancer, also recognized as diffuse-subtype, and found no significant difference in recurrence rate (34.6% vs 15.0%) or 5-year OS (44.0% vs 58.2%) between TG and SG [31]. In an analysis from Gajardo et al. where 130 patients with distal diffuse gastric cancer underwent R0 resection and D2 lymphadenectomy followed by adjuvant chemoradiotherapy, comparable recurrence patterns and 5-year OS (51% vs 63%) were also reported between TG and SG respectively [32]. Finally, Boubaddi et al. evaluated 269 patients with distal diffuse gastric cancer [12], 43.5% of whom completed perioperative FLOT chemotherapy with no differences in 5-year disease-free survival (46.0% vs 45.3%) observed.

In support of these earlier findings, our study was conducted in the FLOT era and found that while TG resulted in a marginally higher median nodal count than SG, this did not reveal a greater burden of positive nodal involvement. Moreover, nodal recurrence was similar between the two surgical approaches, with peritoneal metastases being the predominant site of first recurrence, consistent with the known biological behaviour of diffuse gastric cancer [33]. Although proponents of TG often cite prevention of gastric stump recurrence, this was not substantiated in our cohort, with similar rates of margin-positivity (after multivariate adjustment) and local recurrence between SG and TG. Taken together, these findings support SG as an oncologically safe strategy for distal diffuse gastric cancer.

On univariate analysis, this study found a higher positive proximal margin rate following TG (9%) than SG (4%). Although this may appear counter-intuitive, this is likely explained by imbalances in the location of tumors between the two groups. Whilst all tumors in this study were located > 5 cm below the gastroesophageal junction, there were more tumors located in the gastric body within the TG group than the SG group (Table S2. Notably, after adjusting for tumor location through multivariate analysis, the risk of proximal margin-positivity was comparable between the two groups. Supporting this, the risk of proximal margin-positivity, segregated by tumor location within the stomach, was similar between TG and SG groups (Table S2). Moreover, the overall rate of margin-positivity in this study is in keeping with the FLOT4-AIO trial [14, 15], and other studies examining distal diffuse gastric cancer [30–32].

The observed OS advantage of SG over TG in our cohort is important but difficult to reconcile within the scope of this study. Interestingly, although not statistically significant, a favorable trend in OS was also seen in the aforementioned studies [30–32]. In our cohort, improved OS in the SG group is not easily explained by oncological factors as the two groups exhibited comparable disease staging, perioperative treatment, recurrence patterns and TTR. Baseline differences in lymphovascular and perineural invasion were also accounted for using Cox regression as part of the OS analysis (Table S4). Moreover, patients who underwent TG had similar, perhaps marginally better physiological fitness (being both younger and less comorbid) at diagnosis, than the SG group. This likely excludes pre-existing patient factors as contributors to inferior OS in the TG group. Therefore, we postulate that this discrepancy in OS may reflect long-term morbidity from higher surgical complications and/or functional burden imposed by TG itself [34–37], predisposing patients to chronic malnutrition, sarcopenia, and immunological dysfunction, all of which may compromise quality-of-life and prognosis [38–41]. The higher complication rate following TG may have also contributed to delays in receiving adjuvant chemotherapy, which could affect long-term survival outcomes, though the similar rates of recurrence and TTR between groups suggest this is unlikely to be the sole explanation in the present study. Our findings are corroborated by a recent meta-analysis of 15 studies involving 6,303 patients, which found that SG was associated with a near two-fold improvement in 5-year OS compared to TG [17].

These findings have important implications for current surgical practices, as a gastric-conserving approach appears to have tangible benefits for patients. Compared to TG, SG retains some gastric reservoir capacity, secretory function, and intrinsic regulation of gut hormone signaling, thereby supporting improved nutritional status for patients [42]. Additionally, as demonstrated in this study, there are also notable benefits of reduced perioperative morbidity and hospital resource utilization with SG, a finding that is consistently reported from the international *GASTRODATA* registry [43] and recent meta-analyses [17]. Partial stomach preservation also enhances patient-reported outcomes, with patients undergoing SG recording better scores than TG on multiple validated quality-of-life instruments, persisting for several years postoperatively [18, 44–46].

As the largest series of patients with locally advanced distal diffuse gastric cancer treated in the FLOT chemotherapy era, our cohort provides robust statistical power to detect clinically meaningful differences in patient outcomes. Drawing from data obtained across 43 international centers, these results reflect contemporary real-world practices, thus providing clinically relevant and broadly generalizable insights. Despite these methodological strengths, several limitations warrant consideration. First, the retrospective design may introduce the potential for selection bias. The choice of surgical procedure (TG versus SG) in clinical practice is determined by a combination of institutional policy, surgeon preference, preoperative endoscopic assessment, and intraoperative findings. As a registry-based study, the granularity of this nuanced decision-making was not captured. Moreover, it is not possible to retrospectively and accurately discern the specific combinations of rationale for the extent of resection on a case-by-case basis. This remains an inherent limitation of multicenter retrospective research that is not a randomized controlled trial. Despite this, we found that baseline staging was comparable between groups, and the influence of residual confounders were accounted for through multivariate analysis. We acknowledge that the true size of these diffuse type tumors is difficult to accurately quantify and adjust for. Second, while the multicenter design enhances external validity, it does introduce heterogeneity in perioperative management, intraoperative strategies, and surgical techniques that cannot be fully standardized. Additionally, while the SPACE-FLOT collaboration includes centers with recognized expertise, not all participating institutions may meet specific international definitions of 'high-volume’ gastric cancer centers. The consistently low morbidity and zero perioperative mortality rates, however, underscore the high-quality care across all participating institutions. Third, while hazard ratios are presented for all recurrence sites in Figure S1, those with low events should be interpreted cautiously, as small sample sizes may yield unstable hazard ratio estimates that limit definitive conclusions about site-specific recurrence risks. Fourth, our findings are not applicable to patients with *CDH1* gene mutated or other types of hereditary gastric cancer syndromes where TG would be indicated. Finally, we did not collect patient reported outcome measures or quality-of-life data which would have provided insights into survivorship experiences to complement our OS findings. Future randomized studies comparing TG and SG are warranted, and such trials should integrate longitudinal nutritional monitoring to better understand the mechanisms underlying the observed survival advantage of partial stomach preservation.

## Conclusion

This international cohort study suggests that in appropriately selected patients, SG achieves similar oncological outcomes when compared with TG for distal diffuse gastric cancers while conferring superior OS advantage. These findings challenge the notion that TG should be offered routinely for all distal diffuse gastric cancers.We recommend that multidisciplinary tumor boards incorporate these findings into treatment planning and consider a gastric-conserving approach for distal diffuse stomach cancer when deemed oncologically appropriate. This would reduce patient morbidity and may enhance their quality-of-life.

## Supplementary Information

Below is the link to the electronic supplementary material.

Supplementary Material 1
