# Supplementary material for: Total versus subtotal gastrectomy following neoadjuvant flot chemotherapy for distal diffuse gastric adenocarcinoma: an international cohort study
Source: Gastric Cancer. 2026 Apr 27;29(4):827–40. doi: 10.1007/s10120-026-01746-7 (PMC13314699; doi:10.1007/s10120-026-01746-7)
Supplement: Supplementary file 1 — Supplementary Material 1 [file 10120_2026_1746_MOESM1_ESM.docx]

**SUPPLEMENTARY MATERIALS**

**Supplementary Appendixes**

Appendix 1. Collaborators 4

Appendix 2. Collaborators’ contribution 5

**Supplementary Results**

Table S1. Participating centers 7

Table S2. Proximal margin status and gastric cancer location 8

Table S2. Univariate and multivariate cox regression analysis for disease free survival 9

Table S3. Univariate and multivariate cox regression analysis for overall survival 10

Figure S1. 11

**Appendix 1. Collaborators**

David S. Liu**,** Jonathan Sivakumar, Margaret M. Lee, Katheryn Hall, David I. Watson, Claire L. Donohoe, Darren J. Wong, Cuong P. Duong, Tim Bright, Ahmad Aly, Sonia Gill, Chao Cheng, Su Kah Goh, Matthew Read, James Tan, Sean Stevens, Enoch Wong, Geraldine Ooi, Yick Ho Lam**,** Eunice Lee**,** David Williams, Louise Jackett**,** Lorenzo Ferri, Jimmy So, Kevin Chan, Garett Smith, David L. Chan, Neil Merrett, Sivakumar Gananadha, Harsh Kanhere, Lauren Kennedy, Mark Smithers, Janine Thomas, Michael Bozin, Lynn Chong, Krinal Mori, Mary-Ann Johnson, Sarah A. Martin, Val Usatoff, Rod Jacobs, Yahya Al-Habbal, Chon Hann Liew, Fredrick Huynh, Robert Bohmer, Girish Pande, Jurstine Daruwalla, Mo Ballal, Deanna Lee, Rukshan Ranjan, Andrew D. MacCormick, James Wilkins, Sharon Pattison, Nicholas Evennett, James Wilkins, Jason Robertson, Mark Pang, Alexandra Gordon, Simon Bann, Yu Kai Lim, Inian Samarasam, Ramesh Gurunathan, Jonathan Yeung, Frances Allison, Aya Siblini, Ewen A Griffiths, Alexander W. Phillips, Pooja Prasad, Sheraz Markar, Swathikan Chidambaram, David Chan, Thomas Murphy, John Reynolds, Magnus Nilsson, Fredrik Klevebro, Guillaume Piessen, Justine Lerooy, Bas Wijnhoven, Charlène van der Zijden, Richard van Hillegersberg, Lianne Triemstra, Jelle Ruurda, Mark Ivo van Berge Henegouwen, Suzanne Sarah Gisbertz, Pietro Maria Lombardi, Aleksandra Edmondson, Joe Q. Wei, Aldenb Lorenzo, Sam Alhayo, Aaditya Narendra, Aadil Rahim, Rocita Ho, Jeremy Granger, Steven Tran, Michalis Koullouros, Alain Nguyen, Christina McVeay, Siang Wei Gan, Eve Hopping, Iain Thomson, Andrew Barbour, David Gotley, Adam Frankel, Riteshkumar Patel, Shaun Jin Hui Chew, Kevin Lah, Sonia Gill, Stephen A. Barnett, Vijayaragavan Muralidharan, Samantha Phillips, Wael Jamel, Bung-Kook Ko, Shantanu Joglekar, Ashray Rajagopalan, Joseph Jaya, Yat Cheung Chung, Saania Peeroo, Marek Bak, Jonathan Tiong, Zhei Zhou, Amy Crowe, Ryan Newbold, Bethanie Trainor, Mei Lynn Pac Soo, Vaibhavee Khandelwal, Nicholas Eikelboom, Kyungchul Kim, Emily Moran, Joshua Hammerschlag, Brendan Desmond, Joel D'Souza, Jacky Lu, Rachel McLay-Barnes, Alexandra Gower, Jenny Choi, Yu Kai Lim, Douglas Wood, Kate Whytock, Suraj Surendran, Negine Paul, Feroz Khan H, Daryl K.A. Chia, Eugene KF. Leong, Tvisha Ijner, Yi-Tzu Linda Lin, Mei Sien Liew, Helen Jaretzke, Niall Dempster, Kunal Bhanot, Areeb Mian, Sotiris Mastoridis, Ben Gibbons, Sam Owen-Smith, James Walmsley, Mohammed Al Azzawi, Evin Doyle, Yasuhiro Okamura, Kammy Keywani, Giovanni Ferrari, Monica Gualtierotti, Paolo De Martini, Frida Bushati

**Appendix 2. Collaborator’s contribution**

**Coordinating principal investigator:** David S. Liu

**Manuscript writing committee:** Jonathan Sivakumar, Darren J. Wong, Katheryn Hall, Margaret M. Lee, Cuong P. Duong, David I. Watson, Claire L. Donohoe, Tim Bright, Ahmad Aly, David S. Liu

**Study steering committee:** David S. Liu, Cuong P. Duong, David I. Watson, Tim Bright, Ahmad Aly, Margaret M. Lee

**Study management group:** David S. Liu, Katheryn Hall, Sonia Gill, Chao Cheng, Su Kah Goh, Darren J. Wong, Tim Bright, Cuong P. Duong, David I. Watson, Matthew Read, James Tan, Sean Stevens, Enoch Wong, Geraldine Ooi, Yick Ho Lam

**Study coordinator:** Katheryn Hall

**Biostatistician:** Darren J. Wong

**Database technicians:** Su Kah Goh, Eunice Lee

**Gastrointestinal pathologists:** David Williams, Louise Jackett

**European study lead:** Claire L. Donohoe

**New Zealand study lead:** James Tan

**Hospital leads**

(Royal Brisbane and Women’s Hospital, Australia) Kevin Chan, (Royal Northshore Hospital, Australia) Garett Smith, David L. Chan, (Bankstown Hospital, Australia) Neil Merrett, (Canberra Hospital, Australia) Sivakumar Gananadha, (Flinders Medical Centre, Australia) Tim Bright, David I. Watson, (Lyell McEwin Hospital, Australia) Yick Ho Lam, (Royal Adelaide Hospital, Australia) Harsh Kanhere, Lauren Kennedy, (Princess Alexandra Hospital, Australia) Mark Smithers, Janine Thomas, (Peter MacCallum Cancer Centre, Australia) Cuong Duong, Michael Bozin, (Austin Hospital, Australia) Ahmad Aly, David S. Liu, (St Vincent’s Hospital, Australia) Matthew Read, Lynn Chong, (Northern Hospital, Australia) Krinal Mori, (Box Hill Hospital, Australia) Mary-Ann Johnson, Enoch Wong, Margaret M. Lee, (Monash Medical Centre, Australia) Sarah A. Martin, Geraldine Ooi, (Western Hospital, Australia) Val Usatoff, Rod Jacobs, Yahya Al-Habbal, (Bendigo Hospital, Australia) Chon Hann Liew, Fredrick Huynh, (Royal Hobart Hospital, Australia) Robert Bohmer, (Launceston General Hospital, Australia) Girish Pande, Jurstine Daruwalla, (Fiona Stanley Hospital, Australia) Mo Ballal, Deanna Lee, Enoch Wong, (Christchurch Hospital, New Zealand) Rukshan Ranjan, (Middlemore Hospital, New Zealand) Andrew D. MacCormick, James Wilkins, (Health New Zealand Te Whatu Ora – Southern, New Zealand) Sharon Pattison, (Auckland City Hospital, New Zealand) Nicholas Evennett, James Wilkins, (North Shore Hospital, New Zealand) Jason Robertson, Mark Pang, James Tan, (Palmerston North Hospital, New Zealand) Alexandra Gordon, (Wellington Regional Hospital, New Zealand) Simon Bann, Yu Kai Lim, (Christian Medical College Hospital, India) Inian Samarasam, (CENGILD GI Medical Center, Malaysia) Ramesh Gurunathan, (National University Hospital, National University Health System, Singapore) Jimmy So, (Toronto General Hospital, Canada) Jonathan Yeung, Frances Allison, (Montreal General Hospital, Canada) Lorenzo Ferri, Aya Siblini, (Queen Elizabeth Hospital Birmingham, England) Ewen A Griffiths, (Royal Victoria Infirmary, England) Alexander W Phillips, Pooja Prasad, (Oxford University Hospital, England) Sheraz Markar, Swathikan Chidambaram, (University Hospitals Plymouth, England) David Chan, (Mercy University Hospital, Ireland) Thomas Murphy, (St James Hospital, Ireland) John Reynolds, Claire L. Donohoe, (Karolinska University Hospital and CLINTEC Karolinska Institutet, Stockholm, Sweden) Magnus Nilsson, Fredrik Klevebro, (Lille University Hospital, France) Guillaume Piessen, (Erasmus University Medical Center, Netherlands) Bas Wijnhoven, Charlène van der Zijden, (University Medical Centre Utrecht, Netherlands) Richard van Hillegersberg, Lianne Triemstra, Jelle Ruurda, (Amsterdam UMC, University of Amsterdam, and Cancer Center Amsterdam, Amsterdam, the Netherlands) Mark Ivo van Berge Henegouwen, Suzanne Sarah Gisbertz, (Niguarda Cancer Centre, Italy) Pietro Maria Lombardi

**Investigators**

(Royal Brisbane and Women’s Hospital, Australia) Aleksandra Edmundson, (Royal Northshore Hospital, Australia) Joe Q. Wei, (Bankstown Hospital, Australia) Aldenb Lorenzo, Sam Alhayo, Aaditya Narendra, (Canberra Hospital, Australia) Aadil Rahim, (Flinders Medical Centre, Australia) Yick Ho Lam, Rocita Ho, Jeremy Granger, Steven Tran, Michalis Koullouros, (Lyell McEwin Hospital, Australia) Alain Nguyen, Christina McVeay, (Royal Adelaide Hospital, Australia) Siang Wei Gan, Eve Hopping, (Princess Alexandra Hospital, Australia) Iain Thomson, Andrew Barbour, David Gotley, Adam Frankel, (Peter MacCallum Cancer Centre, Australia) Riteshkumar Patel, Shaun Jin Hui Chew, (Austin Hospital, Australia) Kevin Lah, Sonia Gill, Stephen A. Barnett, Vijayaragavan Muralidharan (St Vincent’s Hospital, Australia) Samantha Phillips, (Northern Hospital, Australia) Wael Jamel, (Box Hill Hospital, Australia) Bung-Kook Ko, Shantanu Joglekar, (Monash Medical Centre, Australia) Ashray Rajagopalan, Joseph Jaya, Yat Cheung Chung, Saania Peeroo, Marek Bak, Jonathan Tiong, (Western Hospital, Australia) Zhei Zhou, (Bendigo Hospital, Australia) Amy Crowe, (Royal Hobart Hospital, Australia) Ryan Newbold, (Launceston General Hospital, Australia) Bethanie Trainor, Mei Lynn Pac Soo, Vaibhavee Khandelwal, (Fiona Stanley Hospital, Australia) Nicholas Eikelboom, Kyungchul Kim, Emily Moran, (Christchurch Hospital, New Zealand) Joshua Hammerschlag, Brendan Desmond, Joel D'Souza, (Middlemore Hospital, New Zealand) Jacky Lu, (Health New Zealand Te Whatu Ora – Southern, New Zealand) Rachel McLay-Barnes, (Auckland City Hospital, New Zealand) Alexandra Gower, (North Shore Hospital, New Zealand) Jenny Choi, (Wellington Regional Hospital, New Zealand) Yu Kai Lim, Douglas Wood, Kate Whytock, (Christian Medical College Hospital, India) Suraj Surendran, Negine Paul, Feroz Khan H, (National University Hospital, National University Health System, Singapore) Daryl K.A. Chia, Eugene Kwong Fei Leong, (Montreal General Hospital, Canada) Tvisha Ijner, (Queen Elizabeth Hospital Birmingham, England) Yi-Tzu Linda Lin, Mei Sien Liew, (Royal Victoria Infirmary, England) Helen Jaretzke, (Oxford University Hospital, England) Niall Dempster, Kunal Bhanot, Areeb Mian, Sotiris Mastoridis, (University Hospitals Plymouth, England) Ben Gibbons, Sam Owen-Smith, James Walmsley, (Mercy University Hospital, Ireland) Mohammed Al Azzawi, (St James Hospital, Ireland) Noel Edward Donlon, Evin Doyle, (Karolinska University Hospital and CLINTEC Karolinska Institutet, Stockholm, Sweden) Yasuhiro Okamura, (Lille University Hospital, France) Julie Veziant, Daniele Leveque, Justine Lerooy, (Amsterdam UMC, University of Amsterdam, and Cancer Center Amsterdam, Amsterdam, the Netherlands) Kammy Keywani, (Niguarda Cancer Centre, Italy) Giovanni Ferrari, Monica Gualtierotti, Paolo De Martini, Frida Bushati

**Table S1.** Participating centers

| **Institute** | **Country** |
| --- | --- |
| Austin Hospital | Australia |
| Bankstown/Liverpool Hospital | Australia |
| Bendigo Hospital | Australia |
| Canberra Hospital | Australia |
| Eastern Health | Australia |
| Fiona Stanley Hospital | Australia |
| Flinders Medical Centre | Australia |
| Launceston General Hospital | Australia |
| Lyell McEwin Hospital | Australia |
| Monash Medical Centre | Australia |
| Northern Health | Australia |
| Peter MacCallum Cancer Centre | Australia |
| Princess Alexandra Hospital | Australia |
| Royal Brisbane and Women’s Hospital | Australia |
| Royal Adelaide Hospital | Australia |
| Royal Hobart Hospital | Australia |
| The Royal Northshore Hospital | Australia |
| St Vincent’s Hospital | Australia |
| Western Health | Australia |
| Auckland City Hospital | New Zealand |
| Christchurch Hospital | New Zealand |
| Dunedin Hospital | New Zealand |
| Middlemore Hospital | New Zealand |
| North Shore Hospital | New Zealand |
| Palmerston North Hospital | New Zealand |
| Wellington Regional Hospital | New Zealand |
| Oxford University Hospital | England |
| Royal Victoria Infirmary | England |
| University Hospital Plymouth | England |
| Queen Elizabeth Hospital Birmingham | England |
| Mercy University Hospital | Ireland |
| St James Hospital | Ireland |
| Karolinska University Hospital | Sweden |
| Lille University Hospital | France |
| Niguarda Hospital | Italy |
| Amsterdam UMC | The Netherlands |
| Erasmus University Medical Center | The Netherlands |
| University Medical Center Utrecht | The Netherlands |
| Christian Medical College Hospital | India |
| CENGILD GI Medical Center | Malaysia |
| National University Hospital | Singapore |
| Toronto University Hospital | Canada |
| Montreal General Hospital | Canada |

**Table S2.** Proximal margin status and gastric cancer location

|  | **Total gastrectomy**  **(n=188)** | | **Subtotal gastrectomy**  **(n=294)** | | **Univariate analysis comparing positive proximal margin rate** | |
| --- | --- | --- | --- | --- | --- | --- |
| **Gastric location** | **Total**  **case number** | **Positive proximal margin** | **Total**  **case number** | **Positive proximal margin** | **OR**  **(95% CI)** | **p-value** |
| Body, n (%) | 143 (76.1) | 17 (11.9) | 86 (29.3) | 7 (8.1) | 1.52 (0.62-3.92) | 0.505 |
| Antrum, n (%) | 38 (20.2) | 0 (0.0) | 168 (57.1) | 2 (1.2) | - | 1.000 |
| Pre-pylorus, n (%) | 7 (3.7) | 0 (0.0) | 40 (13.6) | 2 (5.0) | - | 1.000 |

CI: Confidence interval; OR: Odds ratio


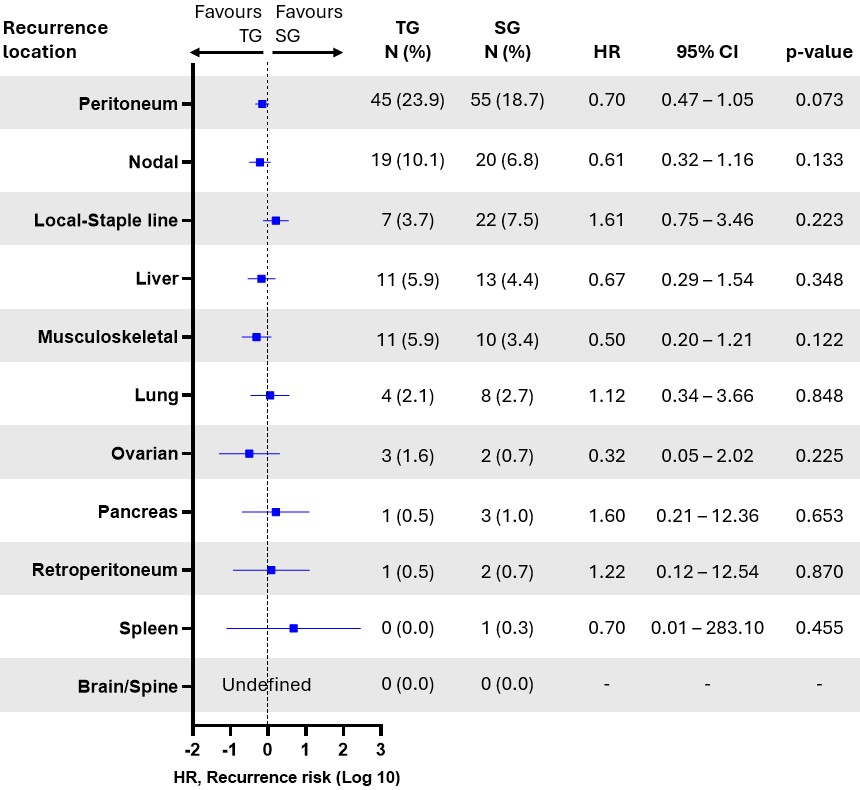


**Figure S1.** Pattern of cancer recurrence in patients with diffuse distal gastric adenocarcinoma who underwent TG versus SG. TG, Total gastrectomy; SG, Subtotal gastrectomy; HR, Hazard ratio; CI, Confidence interval


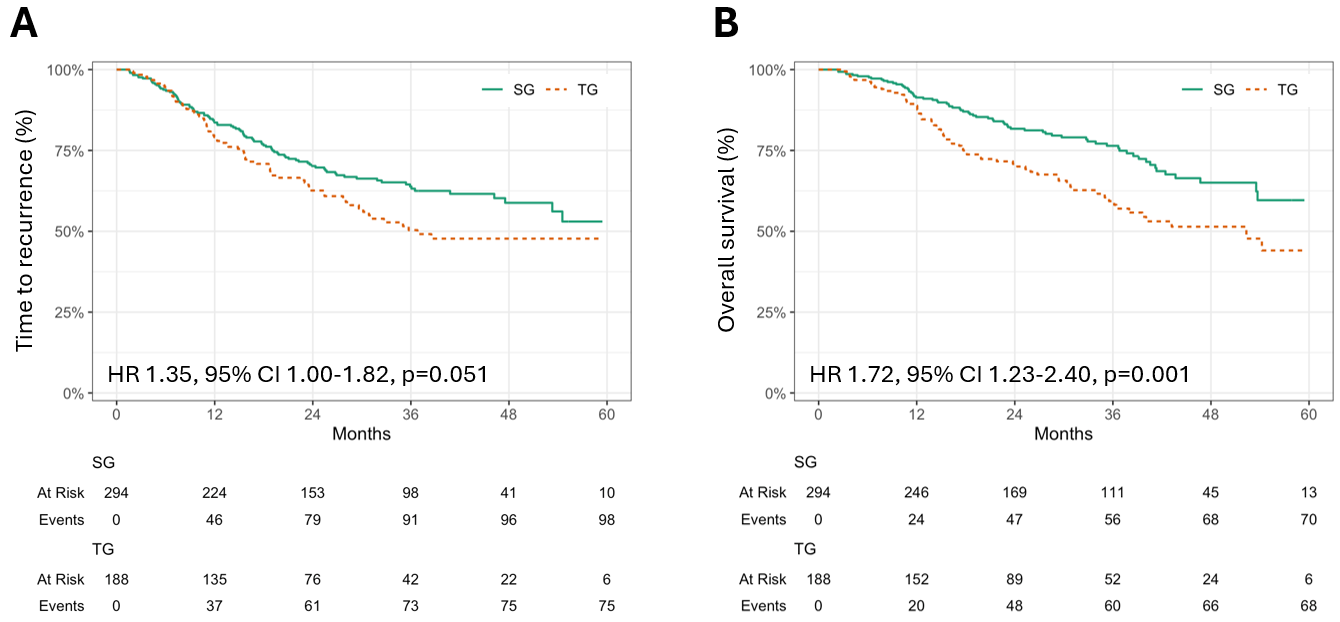


**Figure S2.** Comparison of patients with diffuse distal gastric adenocarcinoma undergoing TG versus SG with respect to (A) unadjusted time to recurrence and (B) overall survival. TG, Total gastrectomy; SG, Subtotal gastrectomy; HR, Hazard ratio; CI, Confidence interval
